# Implementation of collaborative governance in cross-sector innovation and education networks: evidence from the National Health Service in England

**DOI:** 10.1186/s12913-014-0552-y

**Published:** 2014-11-08

**Authors:** Pavel V Ovseiko, Catherine O’Sullivan, Susan C Powell, Stephen M Davies, Alastair M Buchan

**Affiliations:** Medical Sciences Division, University of Oxford, John Radcliffe Hospital, Oxford, OX3 9DU UK; Thames Valley HIEC, Oxford, UK; Manchester Metropolitan University, Manchester, UK; London School of Hygiene and Tropical Medicine, London, UK; Addenbrooke’s Charitable Trust, Cambridge, UK; Oxford University Hospitals NHS Trust, Oxford, UK

**Keywords:** Collaborative governance, National Health Service (NHS), Health Innovation and Education Cluster (HIEC), Network, Partnership

## Abstract

**Background:**

Increasingly, health policy-makers and managers all over the world look for alternative forms of organisation and governance in order to add more value and quality to their health systems. In recent years, the central government in England mandated several cross-sector health initiatives based on collaborative governance arrangements. However, there is little empirical evidence that examines local implementation responses to such centrally-mandated collaborations.

**Methods:**

Data from the national study of Health Innovation and Education Clusters (HIECs) are used to provide comprehensive empirical evidence about the implementation of collaborative governance arrangements in cross-sector health networks in England. The study employed a mixed-methods approach, integrating both quantitative and qualitative data from a national survey of the entire population of HIEC directors (N = 17; response rate = 100%), a group discussion with 7 HIEC directors, and 15 in-depth interviews with HIEC directors and chairs.

**Results:**

The study provides a description and analysis of local implementation responses to the central government mandate to establish HIECs. The latter represent cross-sector health networks characterised by a vague mandate with the provision of a small amount of new resources. Our findings indicate that in the case of HIECs such a mandate resulted in the creation of rather fluid and informal partnerships, which over the period of three years made partial-to-full progress on governance activities and, in most cases, did not become self-sustaining without government funding.

**Conclusion:**

This study has produced valuable insights into the implementation responses in HIECs and possibly other cross-sector collaborations characterised by a vague mandate with the provision of a small amount of new resources. There is little evidence that local dominant coalitions appropriated the central HIEC mandate to their own ends. On the other hand, there is evidence of interpretation and implementation of the central mandate by HIEC leaders to serve their local needs. These findings augur well for Academic Health Science Networks, which pick up the mantle of large-scale, cross-sector collaborations for health and innovation. This study also highlights that a supportive policy environment and sufficient time would be crucial to the successful implementation of new cross-sector health collaborations.

**Electronic supplementary material:**

The online version of this article (doi:10.1186/s12913-014-0552-y) contains supplementary material, which is available to authorized users.

## Background

Increasingly, health policy-makers and managers all over the world look for alternative forms of organisation and governance in order to add more value and quality to their health systems. Network forms of organisation and governance represent an alternative to traditional hierarchies or markets. Their spread can be empirically observed both in the public and private sector in different countries [1]. In the British public sector, they have been invoked as a means of addressing the many social issues that cut across organisation and sector boundaries [2]. In the National Health Service (NHS), “managed clinical networks” were established in the 1990’s, and other forms of network followed [3]. More recently, attention has turned to the anticipated benefits of collaboration with the higher education sector, local authorities, industry, and the third sector. In addition to improvements to the local health systems, policy-makers anticipate that cross-sector collaboration will drive innovation and economic gain. They believe that cross-sector collaborations enable participant organisations to manage large-scale change, to exchange information, leverage each other’s resources, and co-ordinate activities. Yet, evidence from the United States suggests that cross-sector health partnerships may be difficult to implement and govern for the following reasons [4,5]:

- they are based on voluntary collaboration rather than hierarchical control;
- participant organisations have different time horizons, risk orientations, and decision-making styles;
- accountability can be difficult to define and enforce; and
- the levels of commitment and resource participation differ between organisations.

In recent years, the central government in England mandated several cross-sector initiatives aimed at improving the quality of care in the NHS and developing the landscape for innovation [6]. While scholars and practitioners have begun to examine these initiatives [7-27], there is a paucity of evidence about the implementation of collaborative governance arrangements. This article contributes to the literature on collaborative governance in cross-sector health networks by providing comprehensive empirical evidence about collaborative governance arrangements and practices in one such network in England – the Health Innovation and Education Clusters (HIECs). In doing so, the article increases the understanding of collaborative governance in cross-sector networks in England’s publicly funded and run NHS, with the potential for the findings discussed here to be considered within other health systems internationally.

### Cross-sector collaborative governance in the NHS

HIECs were one of several types of formal cross-sector health initiatives based on collaborative governance that were mandated by the Department of Health, England in recent years as part of the NHS innovation landscape (Table 1).

**Table 1:** **Cross-sector collaborations designated by the Department of Health in England, 2008-2014**

	**Designation**	**Partnership composition**	Description of expected activity*	Provision of new resources*
**NIHR Biomedical Research Centres (BRCs)**	2007 for five years;	NHS provider trusts and higher education institutions	“to conduct translational research to transform scientific breakthroughs into life-saving treatments for patients” [29]	5 designated NIHR Comprehensive BRCs and 7 designated NIHR Specialist BRCs received £450 m of government funding for 2007–2012 [28];
	2012 for five years			11 designated NIHR BRCs received £677 m of government funding for 2012–2017, ranging between £2 m and £23 m per NIHR BRC per year [29]
**NIHR Biomedical Research Units (BRUs)**	2008 for four years;	NHS provider trusts and higher education institutions	“to undertake translational research in priority areas of high disease burden and clinical need” [30]	15 designated NIHR BRUs received £55 m of government funding for 2008–2012, up to £1 m per NIHR BRU per year [31];
	2012 for five years			20 designated NIHR BRUs received £126 m of government funding for 2012–2017, ranging between £1 m and £2 m per NIHR BRU per year [30]
**NIHR Collaborations for Leadership in Applied Health Research and Care (CLAHRCs)**	2008 for five years;	NHS providers and commissioners, higher education institutions, and other relevant local organisations	“to conduct applied health research across the NHS, and translate research findings into improved outcomes for patients” [32]	9 designated NIHR CLAHRCs received £50 m of government funding for 2008–2013, ranging between £1 m and £2 m per NIHR CLAHRC per year [28];
	2014 for five years			13 designated NIHR CLAHRCs received £124 m of government funding for 2014–2018, approximately £2 m per NIHR CLAHRC per year [32]
**Academic Health Science Centres (AHSCs)**	2009 for five years;	NHS providers and higher education institutions	“to increase strategic alignment of NHS providers and their university partner, specifically in world-class research, health education and patient care, in order to improve health and healthcare delivery, including through increased translation of discoveries from basic science into benefits for patients” [33]	5 designated AHSCs in 2009 and 6 designated AHSCs in 2014 benefited from the prestige of being designated by an international panel of experts, but were not meant to receive any new government funding [33]
	2014 for five years			
**Health Innovation and Education Clusters (HIECs)**	2010	NHS providers and commissioners, higher education institutions, local government, charities, industry	“to enable high quality patient care and services by quickly bringing the benefits of research and innovation directly to patients, and by strengthening the co-ordination of education and training so that it has the breadth and depth to support excellence” [34]	17 designated HIECs received £11 m of government funding in 2010 and £10 million in 2011, but were meant to become self-sustaining in the longer-term [35]
**Academic Health Science Networks (AHSNs)**	2013 for five years	NHS providers and commissioners, higher education institutions, local government, charities, industry	“to align education, clinical research, informatics, innovation, training and education and healthcare delivery… to improve patient and population health outcomes by translating research into practice, and developing and implementing integrated health care services” [36]	15 designated AHSNs received £70 m of government funding in 2013, ranging between £2 m and £7 m per AHSN per year, and expected to receive further funding for up to five years, but were meant to become self-sustaining in the longer-term [37]

*Characteristics of mandates according to Montjoy RS and O’Toole LJ [38].

In 2007, the National Institute for Health Research (NIHR) mandated and funded new initiatives promoting partnerships for translational research. NIHR funding schemes for Biomedical Research Centres and Units (BRCs and BRUs) invited NHS teaching hospitals and universities to establish formal partnerships for translational research. The NIHR selected the best partnerships through an open competition and provided them with a large amount of funding to conduct translational research [28-31]. An emerging empirical literature has demonstrated a positive impact of NIHR BRCs and NIHR BRUs both on the translational research infrastructure and on the institutional relationships between the NHS, academia, and industry [8,9].

In 2008, a similar NIHR funding scheme for Collaborations for Leadership in Applied Health Research and Care (CLAHRCs) invited NHS teaching hospitals and commissioners, universities, and other relevant local organisations to establish formal partnerships for applied health research. The NIHR selected the best partnerships through an open competition and provided them with a substantial amount of funding to conduct applied health research and translate research findings into practice [28,32]. An emerging empirical literature on NIHR CLAHRCs has successfully applied the community of practice theory to the design and evaluation of theory-informed applied health research, illuminated the boundaries that exist between various professional and organisational groups, and explored ways to mobilise knowledge across such boundaries [13,14,22,27]. Another important strand of the literature on NIHR CLAHRCs has proposed theory-informed realist evaluations to determine “what works, for whom, how, and in what circumstances” [11,19].

In 2009, the Department of Health, England mandated a new kind of partnerships between NHS teaching hospitals and universities – Academic Health Science Centres (AHSCs) [26]. The Department of Health, England selected the best partnerships through an open competition, and provided them with a broad mandate to increase strategic alignment of NHS providers and universities across research, education, and patient care, but without any new resources [33]. Instead, they benefited from the prestige of being recognised by an international panel of experts as the leading partnerships of this kind in England [10]. An emerging empirical literature on AHSCs has analysed the establishment of AHSCs in England within the context of international policy transfer [24], their organisational models [10], organisational culture [17], funding arrangements [25], accountabilities [26], inter-professional dynamics [23], and showed that alignment in AHSCs is hard to achieve because of the bifurcating accountabilities of academic and clinical partners to various government and public agencies [26].

In 2009, the Department of Health, England also mandated wider cross-sector partnerships – Health Innovation and Education Clusters (HIECs). Unlike NIHR BRCs, NIHR BRUs, NIHR CLAHRCs, and AHSCs, which were exclusive partnerships between a single university and its principal NHS affiliates designed to further strengthen centres of excellence in research, HIECs were inclusive partnerships between NHS providers and commissioners, higher education institutions, local government, charities, and industry. The Department of Health, England selected the best partnerships through an open competition, and provided them with a broad mandate to enable high quality patient care by speeding up the adoption and spread of innovation. Seventeen HIECs received a small amount of central government pump-priming funding in the first two years of their existence with the expectation of attracting local match funding and become self-sustaining in the longer run [34,35].

The launch of HIECs coincided with the change of government, and the incoming government proposed a wide-ranging set of reforms, which in the following two years resulted in the dissolution of NHS strategic health authorities, the cessation of funding for the HIECs, and the creation in 2013 of new cross-sector health collaborations, i.e. Academic Health Science Networks (AHSNs). AHSNs included a large number of NHS providers and commissioners, higher education institutions, local authorities, charities, and industry. They received a broad mandate to align education, clinical research, informatics, innovation, training and education, and healthcare delivery in large geographies in order to improve patient and population health outcomes [36,37]. AHSNs received funding towards set-up costs in the first year and expected to receive further funding for up to five years, but were meant to become self-sustaining in the longer-term. Also in 2013, the Department of Health, England decided not to extend the funding for HIECs and the majority ceased to exist in their original form.

### The HIEC mandate

**Figure 1:**
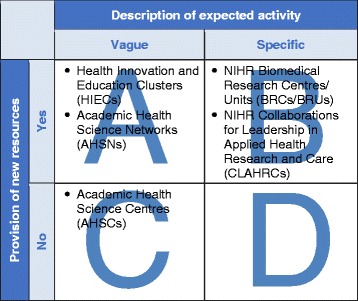
**Cross-sector collaborations designated by the Department of Health in England, 2008–2014: characteristics of mandates according to Montjoy RS and O’Toole LJ[38].**

In order to consider what is distinctive, as well as what is common, about the implementation of collaborative governance in HIECs, it is useful to classify all of the cross-sector health partnerships mentioned above in the same way. For this purpose, we can use a theory-based framework originally proposed by Montjoy and O’Toole for their analysis of problems in intra-organisational implementation [38], which also continue to apply in the context of inter-organisational implementation and are multiplied by the number of participating organisations [39]. The framework distinguishes between two major characteristics of external mandates: (1) whether they are vague or specific, and (2) whether they provide new resources or not (Figure 1) [38]. There are both practical and theoretical advantages of using this framework. For implementation practitioners, it offers a set of assumptions and propositions that highlight potential implementation problems. For implementation scholars, it provides variables that can be used in future research for the development of a more contextualised “programme theory” [19]. For public administration scholars, it provides an opportunity to use our empirical evidence from healthcare to test theory generated through the application of this framework in other domains of public administration.

The framework distinguishes between two major characteristics of external mandates: (1) whether they are vague or specific, and (2) whether they provide new resources or not (Figure 1) [38]. The HIEC mandate is of Type A, i.e. vague with new resources. Such mandates offer the highest degree of discretion to local leaders to interpret and implement the central mandate according to their goals and world-views, using the resources provided. However, such mandates can also result in two potential implementation problems [38]:

- appropriation of the mandate to their own ends by a local dominant coalition (i.e. those who normally get their own way in the direction of activities), and
- inaction due to unwillingness or inability of the local dominant coalition to impose a single interpretation on the mandate.

According to the Department of Health, England, HIECs were intended to be “*partnerships between NHS organisations (primary, secondary and tertiary care), the higher education sector (universities and colleges), industry (healthcare and non-healthcare industries) and other public and private* sector *organisations”* [34]*.* The purpose of HIECs was defined as “*to enable high quality patient care and services by quickly bringing the benefits of research and innovation directly to patients, and by strengthening the co-ordination of education and training so that it has the breadth and depth to support excellence”* [34]. In implementing their mandate, HIECs were expected to adhere to a common set of principles [34]:

- span settings,
- span sectors,
- span professions,
- deliver measurable impact in innovation,
- focus on quality,
- support the commissioner/provider split,
- strengthen accountability.

Governance was central to the implementation of the HIEC mandate because it provided a mechanism for achieving and maintaining a desired boundary-spanning partnership composition, managing competing interests or confusion over the purpose, and providing accountability to the various stakeholders. In a similar way to other cross-sector collaborations (Table 1), the Department of Health, England followed a permissive approach to HIECs’ governance. As a prerequisite for designation, HIECs had to develop robust and effective governance arrangements. In doing so, they were expected to explore various legal forms and determine which governance arrangements best fitted their local needs [34]. Thus, by studying collaborative governance arrangements and practices in HIECs, we can draw lessons for policy-makers who may be considering choices between more or less prescription of forms of governance and specificity of mandate. Moreover, empirical data from HIECs can be used in conjunction with empirical data from other cross-sector collaborations to generalise about cross-sector collaborative governance.

## Methods

This study received approval from the University of Oxford’s Medical Sciences Interdivisional Research Ethics Committee (MSD-IDREC-C1-2012-160). It employed a mixed-methods approach, integrating both quantitative and qualitative data from a national survey, a group discussion, and in-depth interviews. Firstly, we conducted an online survey into Self-Assessment of Governance Arrangements (SAGA) among directors of all 17 HIECs in order to gather both objective data and directors’ perceptions of HIECs’ governance arrangements and practices. The survey achieved a 100% response rate, and thus provided comprehensive information about the entire population of HIECs. Secondly, we held a group discussion with 7 HIEC directors and senior managers to check the validity of survey findings and to identify important collaborative governance issues not covered in the survey. Finally, we conducted in-depth interviews among 10 directors and 5 chairs to triangulate data from the survey and group discussion and to gain broader qualitative insights.

We developed the SAGA survey from a review of the literature on collaborative governance and input from practitioners. The review mainly included US studies, especially a very rich vein of research on collaborative governance in Community Care Networks^SM^ [4,5,40-46]. To formulate an initial pool of questions, we used insights from the relevant literature, and adapted elements from previous studies. These included several concepts and items related to the partnership composition, legal form, decision-making authority, and progress on governance activities [4], decision-making dynamics [45], management [44], accountability [47], and partnership termination and succession [48].

Following feedback from practitioners, we reworked the questions and collated them into an online SAGA survey using SurveyMonkey®. To test its content, readability, and time-to-completion, we piloted the survey with three HIEC directors in December 2012. Following feedback from the pilot, further changes were made to the survey prior to its administration. The final version of the SAGA survey included 83 questions covering 10 substantive categories as well as informed consent, personal details, confidentiality, and willingness to participate in further study (Additional file 1). We fielded the final version of the SAGA survey in January-February 2013, and then analysed its results (Additional file 2).

Two co-authors (CO and PVO) administered the group discussion during a National HIEC Directors’ Network meeting in March 2013. Seven participants were informed of the initial findings of the survey, were asked for their views on the validity and interpretation of the findings, and were guided into a discussion on the important collaborative governance issues not covered in the survey. The group discussion informed both the interpretation of the survey responses and the development of the interview protocol. Two co-authors (PVO and CO) conducted interviews either via telephone or face-to-face. Interview protocols were guided by the survey responses, themes emerging from the group discussion, and by theoretical insights from the relevant literature [38,48-52]. Each interview lasted approximately 30–45 minutes, was digitally recorded and fully transcribed.

Printed interview transcripts (75 pages/36,959 words) and open-ended answers from the survey (7 pages/2,733 words) were coded manually and synthesised around the categories from the survey and new emerging themes. Quotations were used to support and illustrate the quantitative findings and new emerging themes from the survey. Two co-authors (PVO and CO) met regularly to discuss and agree the interpretation and synthesis of the data. To ensure internal consistency, one co-author (SCP) cross-checked the accuracy of the quantitative results of the survey, the interpretation of the qualitative data, and the synthesis of the quantitative and qualitative data.

## Results^a^

### Partnership composition

HIECs were fluid and inclusive networks, with varying degrees of participation of partners from different sectors. Although the term “cluster” implied the close geographical proximity of cluster members, HIECs included partners from large geographical areas. Seventeen HIECs covered 9 of 10 NHS areas in England, and often replicated the boundaries of NHS strategic health authorities, which supported the development and implementation of HIECs. Therefore, HIECs are better understood as geographically dispersed networks, rather than geographically concentrated clusters.

Approximately half of HIECs (53%) had a fixed number of partners, which varied greatly, from 5 to 60, averaging 24 partners. Those HIECs that did not have a fixed number of partners explained this by the fact that HIECs were project-driven networks, and thus different partners participated in different projects. As one respondent put it: *“We aimed to find the right mix of organisations to further our objectives for each project we started”.*

The majority of partners in HIECs were either self-selected foundational members (47%), who all came together to form HIECs, or those invited to join in by the foundational members (47%). In just one HIEC (6%), the majority of the partners were required to apply formally for membership; and none of the HIECs granted membership on a basis of paying a membership fee. Approximately one third of HIECs (29%) had different classes of partners, such as full/core members and affiliates. In the majority of cases, affiliates were those members who elected not to pay a membership fee in HIECs where such a fee existed, but still wanted to participate in HIEC activities. Therefore, having different classes of partners was a strategy aimed at including more partners in HIEC activities.

**Figure 2:**
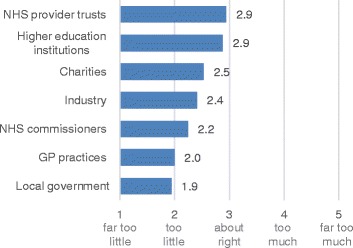
**Cross-sector participation in HIEC activities.**

Participation in collaborative projects across the lifetime of the HIEC varied between different sectors. According to responses from 15 HIECs, all of them had NHS provider trusts, NHS commissioners, and higher education institutions (HEIs) involved in their collaborative projects, on average 10 NHS provider trusts, 4 NHS commissioners, and 5 HEIs. Participation from other sectors varied. When asked to indicate whether participation from different sectors has been sufficient for HIECs to accomplish their objectives, participation from local government, GP practices, and industry was deemed to be too little (Figure 2). One respondent commented: “*Given the scale of some of these sectors, we have done no more than scratch the surface.*”

### Legal form

None of the HIECs were constituted as incorporated bodies and none were registered as a charity. The perception of the limited lifespan and uncertain funding of HIECs due to political instability was cited by the respondents as the main reason for not seeking incorporation or registration. One respondent remarked: “*It felt like the environment was sufficiently unstable, that we should use our funding and position to start work, and form a bridge to whatever would happen next*”. Other major reasons included the complexity and cost of creating a corporate legal entity for such a diverse number of partners from different sectors, and a lack of perceived benefits. Likewise, only 41% of HIECs have a signed membership agreement, or a memorandum of understanding.

Although HIECs did not have their own legal personality, they were hosted by legal entities, mostly by HEIs and NHS provider trusts, or as in one case a corporate legal entity created by a HEI and NHS provider trusts, i.e. an Academic Health Science Centre. Importantly, many respondents felt that not having to deal with incorporation or registration as a charity speeded up the implementation of HIECs. The initial governance arrangements of HIECs had to be sufficiently robust to satisfy the host organisation, but we did not find any evidence to suggest that they assimilated governance practices from their host organisation. Whereas the perceived benefits of being hosted by a HEI included flexibility, entrepreneurship, and stability, the perceived benefits of being hosted by an NHS provider trust included credibility with NHS partners, ownership within the NHS, and allowing staff to retain NHS pension rights. All HIECs valued operational independence from their host organisation, and there were no significant differences identified in the nature of the hosting arrangements by virtue of HIECs being hosted in different sectors.

Despite the fact that none of the HIECs had a legal personality of their own, in the majority of cases, they nevertheless aimed to emulate the governance processes of formal public sector organisations and were able to persuade a number of senior leaders from the local health system to participate in governance activity throughout the life of the HIEC. One respondent noted: *“We do not actually exist as a legal entity, so we really took the view right from the beginning that it was therefore important to have proper governance and to be seen to be governed properly because that was the only thing we had…it was the only thing to show that we actually existed.”*

### Governing body

HIECs had relatively informal governance arrangements determined by the local circumstances. Most commonly, the governing body of HIECs was called “a board”, “a partnership board”, or “a steering group”, and met bi-monthly or quarterly. It was usually inclusive and, while having no legal constitution, most had a set of Terms of Reference. The number of members on the board was determined by the local circumstances and varied greatly between 5 and 40, averaging 14 members. Only 24% of HIECs made a distinction between non-executive and executive members, and in 18% of HIECs that had membership fees, only paying members had voting rights. Although participation in governance activities for non-voting and non-fee paying members was limited, they still were able to participate in HIEC projects.

There seemed to be no ideal number of board members as only one HIEC that had 15 members on its board indicated that it was too many because of difficulties in meeting quorum requirements. Likewise, there was no ideal breakdown of members by sector. NHS provider trusts, higher education institutions, and NHS commissioners were the most frequently represented sectors on the board; industry, charities, local government, and GP practices were the least frequently represented. Additionally, 41% of HIECs have other bodies or individuals involved in the governance process, e.g. a strategic health authority, a stakeholder group, or a patient representative. Some of the HIECs expressed regret in hindsight that their boards had not been more inclusive: *“Part of the vision was to bring NHS and academic partners together…I accept that the HIEC may also be involved in bringing in industry and so forth, and frankly, I think, we failed comprehensively in that regard.”*

All HIEC governing bodies had a chair, chosen either as an independent person (53%), or a representative of one of partners (47%). In the majority of HIECs, chairs were nominated by key partners, or appointed by consensus, or a vote among all partners. Only one HIEC chair was appointed through an open competitive process. The term of the chair ranged from 1 year to 4 years, or was not specified due to the uncertain lifespan of HIECs. None of the HIEC chairs worked a set number of hours per week, and only 24% of them were paid.

All the interviewed respondents felt that the permissive approach in the HIEC mandate to forming governance arrangements was helpful because it allowed HIECs to develop governance arrangements that suited their local circumstances best, often starting with local enthusiasts for the HIEC vision. Several respondents commented that the Department of Health should not have prescribed a geographical footprint for HIECs because it initially slowed down the formation of HIECs. Most HIECs would probably agree with the chair who commented: “*I personally was of the view that the HIEC should be created as a local mechanism in line with local needs and opportunities, and the only things that should be prescriptive were to be sure that it does make a difference.”*

### Decision-making authority and dynamics

HIEC governing bodies exercised largely independent decision-making authority within their mandate. All HIEC governing bodies had authority to allocate resources and, in the majority of cases, HIEC governing bodies had authority to establish partnership initiatives, as well as to report partnership performance. Yet, in 29% of HIECs, the governing body itself did not have authority to navigate the future through transition. Decision-making in HIECs usually occurred in a non-confrontational, even passive, atmosphere. Only 12% of HIECs felt that decision-making occurred in a politically-charged atmosphere. Moreover, 59% of HIECs felt that they had never had disagreements between or among governing body members. Most respondents believed that this was because HIECs had relatively small budgets.

We did not find evidence, in any of the localities, of a dominant coalition of partners that appropriated the HIEC mandate and resources to their own ends. In some cases, the original group who developed the HIEC bid to the Department of Health might have acted initially as though they were dominant, but that changed once the HIEC came into being. For example, one respondent commented: “*we had three universities, who all contributed in the pre-bid stage to identifying priorities…so the first thing we had to do from the NHS perspective was to say ‘OK, well, we’ll honour that, but we’ve also now got more than half the cake, which now needs to be made available to other [partners]’.”*

This lack of a permanent dominant local coalition might be attributable to the combination of two factors. On the one hand, the HIEC mandate envisaged establishing inclusive partnerships, which did not allow a small number of partners to form strong dominant and exclusive coalitions. On the other hand, the HIEC mandate came with a small amount of new resources, which did not provide sufficient incentive for strong coalitions to emerge.

### Progress on governance activities

**Figure 3:**
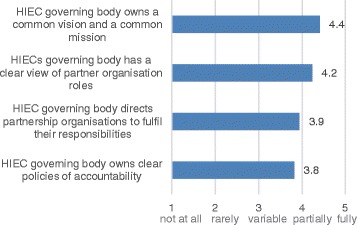
**Progress on governance activities.**

HIECs made partial-to-full progress on governance activities (Figure 3). In 41% of HIECs, the governing body owned a common vision and a common mission only to a partial or variable extent. Most respondents believed that a greater clarity of the HIEC mandate would have helped avoid early-stage delays in implementation due to a prolonged deliberation of the HIEC vision and mission. One respondent recalled: “…*every single member of the HIEC Board had a completely different view of what the HIEC was and what it wanted to achieve. Some of them were quite benign and open, but others, their translation of that was pretty much in terms of what they wanted to get out of that. And it was not really that helpful in terms of the Board coming together*.” In contrast, another respondent noted that, despite delays in implementation, a prolonged deliberation of the HIEC mandate was helpful in the long run because it ensured a lot of buy-in from partners.

In 59% of HIECs, the governing body had a clear view of partner organisation roles only to a partial or variable extent. This lack of full clarity on partner organisation roles was attributed not only to the vague mandate, but also to the intrinsically challenging nature of cross-sector working. One respondent remarked: *“Because our Board is drawn from different sectors, they have some differences in terms of how they conceptualise partnership working.*” In 53% of HIECs, the governing body did not fully direct partnership organisations to fulfil their responsibilities. Respondents attributed this to the fact that HIECs relied on voluntary participation of partner organisations with a varying degree of engagement and commitment.

Accountability was the most challenging area in governance activities. Only 35% of HIECs felt that their governing body fully owned clear policies of accountability. Many respondents believed that it was particularly challenging to manage competing accountabilities of HIEC partners. One respondent noted: “*Our projects have many partners, who may themselves have different accountabilities and even different ideas about what accountability means.*”

### Management

**Figure 4:**
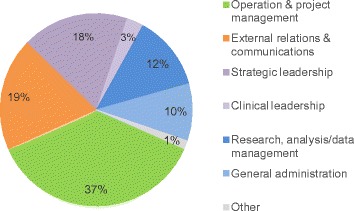
**Breakdown of the overall staff time resource by function.**

HIECs had lean management teams. All HIECs had some paid staff, on average 3.7 whole time equivalent (wte), ranging from 0.2 wte to 9.9 wte. Most staff members were either employed by the host organisation or seconded from a partner organisation. The overall time resource of HIEC staff was spent predominantly on operation and project management, external relations and communication, and strategic leadership (Figure 4).

All HIECs had a chief executive, who was usually called “Director” and sometimes “Chief Executive”. In contrast with HIEC chairs, 53% of HIEC chief executives were appointed through an open competitive process. In many HIECs, chief executives had more experience of governance than chairs. One respondent recalled: “*One of the things I was specifically recruited for was experience of governance. As Chief Executive, I have had considerable influence over the way the Board has evolved and, especially, the way it is drawn from a range of stakeholders.”*

The majority of respondents acknowledged that the relationships between the chief executive and the board were different from those in traditional organisations. Most commonly, the board and the chief executive collaborated in formulating the overall strategy and the board supported the executive in the consequential development of plans and projects to deliver the strategy. This could potentially be construed as the muddying the boundaries between the board and the management. However, there were other mechanisms that secured the scrutiny function. One respondent explained: *“In our HIEC… the way we mitigated any problems was that the chair and the director worked very closely together and, secondly, we did make sure that the SHA [strategic health authority] as the final accountable organisation was comfortable with what we were trying to do, and we regularly reported to them.”*

### Funding

HIECs largely relied on public funding and partners did not commit a significant amount of their own resources. Each HIEC received on average £1.2 m from the Department of Health, England in funding during the first two years in operation. In addition, each HIEC raised on average £0.5 m in funding from other sources. Whereas NHS strategic health authorities and non-NHS sources were the most frequently cited sources of additional funding, membership fees from HIEC members and project funding from HIEC partners were the least frequently cited sources of additional funding. The overall expenditure of HIECs was split between the commissioning of activities through grants or contracts (39%), direct delivery of activities (35%), and facilitation, networking, communication and enabling (25%).

While all HIECs commented on the very small amount of new public resources that was available to them, only 36% of HIECs raised project funding from their partners, and only 21% of HIECs raised funding through membership fees. The latter were seen as a means of securing both tangible commitment as well as funds. The main reasons for not seeking membership fees were: reducing barriers to participation; avoiding duplicating other fee paying networks; and the difficulty of persuading partners to pay up front without proof of concept of the HIEC model.

After three years in operation, only 13% of HIECs reported that they were in a position to be self-sustaining without government funding. Whereas some respondents thought that without substantial funding from the Department of Health, England or other NHS sources, the HIEC model was never going to be self-sustaining, others believed that the structural and political changes in the NHS did not give a chance for HIECs to become self-sustaining. One respondent noted: “*I think we could have been self-sustaining if the political will hadn’t changed, so we were not given the planned third year of funds, and a sufficient time frame to build our profile.”*

### Accountability

HIECs encountered significant external and internal accountability challenges. Whereas HIECs regularly reported on performance to their partners, host organisation, and to their NHS strategic health authority, they rarely reported directly to patients and infrequently to the Department of Health, England. Although one HIEC had a patient representative appointed as a chair of its board and several other HIECs experimented with having a patient representative on their boards, 41% of HIECs never reported to patients. The main reason for this was that NHS partners had their own mechanisms for accountability to patients. Additionally, many HIECs involved patients in their project management.

HIECs expected the Department of Health, England to play a more meaningful role in holding them to account for public money. Many respondents believed that the Department of Health, England should have articulated a vision for HIECs more clearly, possibly, including a set of short-term, medium-term, and long-term metrics or indicators to measure their performance against a set of locally-agreed objectives in a number of priority areas. One respondent in particular felt that effectively there was an “*abdication of responsibility*” on part of the Department of Health, England in not measuring and evaluating the performance of HIECs at the national level.

The majority of HIECs were also critical about the quality of their reporting relationships with NHS strategic health authorities because, as one respondent put it, they often were “*completely tokenistic*.” Some strategic health authorities requested progress reports too frequently, but concentrated mainly on the financial indicators and did not scrutinise the contents of HIEC projects. Those strategic health authorities that developed key performance indicators for HIECs did not actually performance manage HIECs, as one respondent noted, “*there were no consequences if these [indicators] were not adhered to or delivered*.” Finally, a number of HIECs proactively reported to strategic health authorities, but often the latter were not actively interested in HIECs’ reporting because strategic health authorities were in the process of being dissolved.

**Figure 5:**
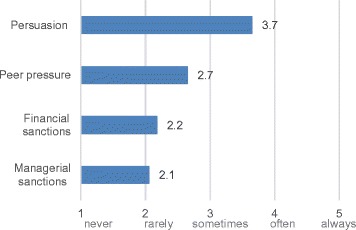
**Frequency of use of different means of enforcement and sanctions for non-performance.**

Nevertheless, HIECs aimed to achieve internal accountability for their projects. Almost always, projects were performance-monitored by the project leads, the governing body, and very often-to-always by the HIEC chief executive. The fact that HIECs were not legal entities limited the scope of means that they could use to enforce accountability. Namely, HIECs could not draw legally-binding contracts themselves and instead had to rely on their host or partner organisations to draw contracts on their behalf. Therefore, when others were delivering on their behalf, HIECs used documented project plans and grant agreements far more frequently than contracts. Likewise, HIECs used persuasion and peer pressure as means of enforcement and sanctions for non-performance far more frequently than financial or managerial sanctions (Figure 5). The use of these forms of sanction should not be interpreted as meaning that accountability was not taken seriously, as several respondents talked about their responsibility to manage public resources wisely. Rather, the fact that HIECs were hosted by other organisation shaped the ways in which they managed accountability relationships.

### Partnership termination, succession, and legacy

HIECs had to deal with the issues of termination, succession, and legacy early in their existence. As one respondent noted: “*We are really just coming into our powers as we are being disbanded*.” Although the funding from the Department of Health, England stopped at the end of year two, HIECs carried on their activities into year three, and some HIECs had planned projects running well into year four. After three years, 13% of HIECs were no longer in operation and 18% more had decided on the termination of their HIEC partnership in their original form. Yet, only 18% of HIECs had decided to continue their partnership in a different form. The main reasons for HIECs’ ceasing operations in their original form were that they became time-limited initiatives due to structural changes in the NHS, and because government mandated new cross-sector networks, AHSNs, which either invited HIECs to join, or replaced their functions.

During their lifespan, 17 HIECs were responsible for over 200 projects, a majority of which focussed on the spread of clinical or managerial innovation or evidence-based practice, particularly, in multi-professional workforce development, integrated care network development, and self-care development for patients [16]. Importantly, HIECs saw their principal legacy as being not only the completion of a range of timely innovation and education projects, but also as having built capacity for collaborative working in their local health economies. Unlike the exclusive cross-sector partnerships between elite teaching hospitals and higher education institutions that predated them, HIECs were inclusive and covered nearly all of England. In many areas, HIECs were the first regional cross-sector health partnerships and thus created a precedent of inter-organisational and cross-sector collaboration. Almost all of the HIECs that were interviewed demonstrated strong examples of collaboration between organisations that were more accustomed to competing, especially teaching hospitals with local district general hospitals. In doing so, many HIECs believed that they had developed in their local economies the capacity for collaborative working that would be particularly useful to the AHSNs.

## Discussion

The aim of our study was to provide comprehensive empirical evidence about the implementation of collaborative governance arrangements in response to the central government mandate to establish HIECs. We conducted a national survey of the entire population of HIEC directors and achieved a 100% response rate. In order to check the validity of survey findings and to gain broader qualitative insights, we conducted a group discussion and in-depth interviews. To the best of our knowledge, this is the first comprehensive study of governance arrangements among all the cross-sector health collaborations mandated by the Department of Health, England in recent years. Because our study is informed by a theory-based framework and generalisable to the entire population of HIECs, our findings provide both theoretically-informed and empirically-tested contributions to the literature on collaborative governance. Our findings will be most relevant to other cross-sector health collaborations characterised by a vague mandate with the provision of a small amount of new resources, but also, to a lesser extent, to other cross-sector collaborations for health. Below, we discuss how the key characteristics of the HIEC mandate and the policy environment shaped local implementation responses and make suggestions for practitioners and policy-makers.

First, local leaders appreciated the opportunity to interpret and implement the HIEC mandate according to their goals and world-views. There was a general consensus among HIECs that the vagueness of the mandate in terms of the partnership composition and governance arrangements positively influenced implementation because it allowed partners to shape HIECs to fit their local needs and circumstances. We found a great variation between HIECs in the number of partners, sector representation, participation, as well as governance arrangements. We also found that the prescription of a geographical footprint for HIECs initially slowed down their formation in some cases. Therefore, we can speculate that a more prescriptive approach to partnership composition and governance arrangements might have resulted in significant implementation delays or even inaction. We suggest that when contemplating the use of vague mandates in terms of partnership composition and governance arrangements to speed up implementation, policy-makers need to take into account the likely variation between partnerships that will arise. This may or may not be of consequence for the policy-maker, depending on their exact policy objectives.

Second, none of the HIECs were incorporated and/or registered as a charity because of the unstable policy environment and the absence of perceived benefits. We did not find evidence that not having their own legal personality adversely affected the implementation of HIECs. On the contrary, many respondents felt that not having to deal with complex legal matters speeded up the implementation of HIECs. The form of the governance model was seen as being of less importance than the idea of the governance function. Our data show that HIEC governing bodies exercised largely independent decision-making authority within their mandate, and that they placed a high value on the governance function in terms of creating a sense of common purpose and collaborative action. It is therefore unclear whether in a more stable policy environment HIECs would have sought incorporation or registration as a charity; or what the benefits of such a move might have been. We propose that further research examines AHSCs and AHSNs that have been successfully incorporated or registered as a charity in order to elucidate the benefits of these legal forms for other cross-sector health partnerships.

Third, our data indicate that progress on governance activities was slowed by the vagueness of the HIEC mandate as well as by the intrinsically challenging nature of cross-sector collaborative working. In some cases, we found evidence of early-stage delays in implementation due to a prolonged deliberation of the HIEC vision and mission. Also, achieving full progress on governance activities was intrinsically challenging due to the very different expectations, engagement, and commitment of the board members who came from different sectors. In line with the findings from the United States that accountability in cross-sector health partnerships can be difficult to define and enforce [4,5], accountability in HIECs proved to be one of the more problematic areas of governance activities. Some respondents believed that the HIEC mandate should have included metrics or indicators to measure HIECs’ performance against a set of locally-agreed objectives in a number of priority areas. This would have facilitated a formal evaluation of the HIECs nationally. We propose that for cross-sector health partnerships with a vague mandate and the provision of new resources, policy-makers develop performance metrics in agreement with each locally-constituted partnership and conduct formal evaluations of their activity at the end of their mandate or funding cycle.

Fourth, in response to the provision of a small amount of new resources, HIECs established lean management teams, which, in most cases, did not become self-sustaining. There were perceptions that the HIEC model was never going to be self-sustaining without some level of government funding, as well as data showing that only a minority of HIECs raised funding from their partners. On the other hand, there were perceptions that HIECs were not given sufficient time to become self-sustaining. We found that after two years of initial government funding, most HIECs carried on their activities into year three, and 13% of HIECs reported that they were in a position to be self-sustaining without government funding. We can conclude that two years of government pump-priming funding was not sufficient for HIECs to become self-sustaining, and that it is likely that over a longer period of time more HIECs would have become self-sustaining. We argue that for cross-sector health partnership that are envisaged to become self-sustaining policy-makers need to provide a longer-term mandate and pump-prime funding.

Fifth, we did not find evidence, in any of the localities, of a permanent dominant coalition of partners that appropriated the HIEC mandate and resources to their own ends. Although we found that self-selected foundational partners had influence on the partnership composition and on key appointments, there is no evidence to suggest that they interpreted the HIEC mandate in their own interests. Likewise, chief executives often had influence on both the strategy and operations of HIECs, but approximately half of them were appointed through an open competitive process, and in many cases they sat on the board. It may have been the case that a strong coalition established the HIEC initially and developed the bid for funding to the Department of Health, England, but in those cases, we found that the HIEC once it was established worked hard to extend the partnership beyond the initial group. It is probably due to HIECs being envisaged as broad cross-sector collaborations, and the fact that only a small amount of new resources was provided, that dominant coalitions of partners did not emerge to appropriate the HIEC mandate and resources to their own ends. We hypothesise that the larger the cross-sector collaboration and the smaller the amount of the new resources provided, the less the probability of local dominant coalitions emerging to appropriate the mandate and resources to their own ends.

Sixth, a broad and inclusive vision for HIECs may have helped to build a capacity for collaborative working. HIECs included a large number of partners and in many areas created a precedent of inter-organisational and cross-sector collaboration. The overwhelming majority of HIECs were able to demonstrate strong examples of collaboration between organisations that were more used to competing. Previous research on Collaboration for Leadership in Applied Health Research and Care (CLAHRCs) showed that “[h]istory appears to be a crucial precursor to more rapid progress within implementation as relationships and ways of working have been developed and tested” [19]. Therefore, we expect that the legacy of collaborative working within HIECs could positively influence implementation of Academic Health Science Networks (AHSNs) – the new cross-sector health networks characterised by a vague mandate with the provision of a small amount of new resources.

Finally, the development of the SAGA survey instrument and the application of the Montjoy and O’Toole framework in the context of cross-sector health partnerships in England represent valuable methodological contributions, which can be used in future research. The SAGA survey instrument allows for rapid data gathering and analysis based on a standardised set of governance concepts and indicators. It can be developed further and applied to study collaborative governance in other cross-sector health partnerships, most notably, AHSNs. The use of the Montjoy and O’Toole framework has a potential to complement the methodological tools used in a growing body of literature on knowledge translation in cross-sector collaborations. The framework draws attention to the process of interpretation of the government mandate by local leaders, which has been shown to explain variation in differing capabilities for knowledge translation among NIHR CLAHRCs [20,22,27]. Furthermore, the framework has a potential to promote organisational learning between different cross-sector health partnerships by identifying partnerships with similar mandate characteristics. For example, in addition to the relevance of findings from HIECs to AHSNs, the framework highlights that findings from NIHR CLAHRCs will be most relevant to NIHR BRCs and NIHR BRUs, which are characterised by a specific mandate with the provision of new resources (Figure 1).

### Limitations

Despite its significant strengths, our study has several limitations. It is based on the perceptions of and data supplied by HIEC directors and chairs who may be biased. Surveying and interviewing the entire population of HIEC board members or partners might have yielded different results. Another limitation is that although the Montjoy and O’Toole framework is a useful tool when comparing the variations in local responses to a central government mandate, it was originally developed for the analysis of intra-organisational implementation. In the context of inter-organisational implementation, impediments to intra-organisational implementation continue to apply and are multiplied by the number of participating organisations, but there may be other impediments as well [38,39]. Therefore, our analysis may not fully reflect the complexity of inter-organisational implementation in cross-sector health collaborations. Yet another limitation is that, in order to reduce the complexity of the mandate characteristics under investigation for analytical purposes, the Montjoy and O’Toole framework represents mandate characteristics as yes/no dichotomies. However, as demonstrated in Table 1, both the description of expected activity and the provision of new resources vary between different partnerships and therefore can be better represented as continuous variables. Finally, we are unable to determine which governance characteristics are associated with greater performance outcomes because there has been no formal evaluation of HIECs’ performance nationally.

## Conclusion

This study has produced valuable insights into the implementation responses in HIECs and possibly other cross-sector collaborations characterised by a vague mandate with the provision of a small amount of new resources. Although, theoretically, there was a risk that a vague mandate with the provision of new resources would lead to local dominant coalitions appropriating the HIEC mandate and resources to their own ends, there is little evidence that such local dominant coalitions emerged. On the other hand, there is evidence of interpretation and implementation of the central mandate by HIEC leaders to serve their local needs. We, therefore, suggest that policy-makers provide a longer-term mandate and funding for cross-sector health partnership that are expected to become self-sustaining. Whereas the vagueness of the mandate in terms of the partnership composition and governance arrangements positively influence implementation, the vagueness of the vision and mission negatively affect progress on governance activities. Accountability proved to be one of the more problematic areas of governance activities. Two years of government funding was not sufficient for HIECs to become self-sustaining and they were adversely affected by an unstable policy environment. A supportive policy environment and sufficient time would be crucial to the successful implementation of new cross-sector collaborations. These findings augur well for AHSNs, which pick up the mantle of large-scale, cross-sector collaborations for health and innovation. We advocate further research to help analyse comparatively the influence of different governance characteristics on performance outcomes in cross-sector health collaborations in order to determine “what works, for whom, how, and in what circumstances” [11,19]; and hope that our work can form a foundation for examining the impact of governance on collaborative working in future.

### Endnote

^a^The preliminary results of this study have been presented at the 1^st^ International Conference of BioMed Central on “Health Services Research: Evidence-based Practice”, London, 1-3, July 2014 (http://www.biomedcentral.com/1472-6963/14/S2/P91).

## Additional files

Additional file 1:
**Survey questionnaire.**
Additional file 2:
**Survey results.**
