# Supplementary material for: Implementation of collaborative governance in cross-sector innovation and education networks: evidence from the National Health Service in England
Source: BMC Health Serv Res. 2014 Nov 8;14:552. doi: 10.1186/s12913-014-0552-y (PMC4263053; doi:10.1186/s12913-014-0552-y)
Supplement: Additional file 1: — Survey questionnaire. [file 12913_2014_552_MOESM1_ESM.pdf]

Additional file 1: Survey questionnaire

Self-Assessment of Governance Arrangements

Consent & personal details

***1. I consent voluntarily to the use of the information and data I provide in the understanding that it will be analysed and presented in a report to the National HIEC Directors Network and then disseminated further. My consent is upon the understanding that all information I provide will be aggregated for purposes of analysis so that my HIEC will not be identified or identifiable, unless I grant permission to cite examples from my HIEC. The information and data I provide will be available on request for verification and will be stored in accordance with the provisions of the Data Protection Act.**

Yes, I consent	No, I do not consent
○	○

***2. Please indicate your name, and if you wish to receive a copy of the report, your email:**

***3. Please indicate your HIEC**

- ○ Cambridgeshire & Peterborough
- ○ East Midlands
- ○ Greater Manchester
- ○ Lancashire and Cumbria
- ○ Norfolk and Suffolk
- ○ North East
- ○ NECLES
- ○ Merseyside and Cheshire
- ○ North West London
- ○ South London
- ○ South West
- ○ Thames Valley
- ○ Wessex
- ○ West Midlands Central
- ○ West Midlands North
- ○ West Midlands South
- ○ Yorkshire and Humber

Partnership composition

***4. Does your HIEC have a fixed number of partners?**

yes	no
○	○

If so, how many?

*5. How have the majority of your HIEC partners been selected?

- ◯ self-selected (foundational members)
- ◯ by request
- ◯ by invitation
- ◯ by paying a fee
- ◯ other (please specify)

6. Does your HIEC have different classes of partners, e.g. full and affiliates?

	yes	no
	◯	◯

If so, how many of each? Please explain the rationale for having different classes of partners.

7. Across the lifetime of your HIEC, please estimate approximately how many different organisations from each of these sectors have participated in collaborative projects initiated and supported through your HIEC. If impossible to estimate, please proceed to the next question.

NHS provider trusts

NHS commissioners

GP practices

higher education institutions

industry

charities

local government

other (please specify)

*8. Across the lifetime of your HIEC, please indicate whether you feel that participation from each of these sectors has been sufficient for your HIEC to accomplish its objectives.

	far too little	too little	about right	too much	far too much
NHS provider trusts	◯	◯	◯	◯	◯
NHS commissioners	◯	◯	◯	◯	◯
GP practices	◯	◯	◯	◯	◯
higher education institutions	◯	◯	◯	◯	◯
industry	◯	◯	◯	◯	◯
charities	◯	◯	◯	◯	◯
local government	◯	◯	◯	◯	◯

If other than about right, please explain

9. Please add any further comments you may have about the composition of your partnership.

Legal form

***10. Does your HIEC have a signed membership agreement or memorandum of understanding?**

yes	no
○	○

If so, is it legally-binding? Please explain its purpose.

***11. Is your HIEC registered as a charity?**

yes	no
○	○

If so, which type of charity? Please explain the benefits of charitable registration.

***12. What is the incorporation status of your HIEC?**

○ incorporated, e.g. a company limited by guarantee

○ unincorporated, e.g hosted by an NHS trust or university

If incorporated

13. Which form of legal entity?

14. What are the benefits of incorporation?

15. Please add any further comments you may have about your HIEC's legal form.

If unincorporated

16. What are the reasons for not seeking incorporation?

17. Which sector does your HIEC's host organisation represent?

- ◯ NHS provider trust
- ◯ NHS commissioners
- ◯ GP practices
- ◯ higher education institutions
- ◯ industry
- ◯ charities
- ◯ local government
- ◯ other (please specify); please also use this box to explain if your HIEC has any form of co-hosting, e.g. one organisation holds the funds and another one employs the staff

18. What are the benefits of your HIEC's hosting arrangements?

19. Please add any further comments you may have about your HIEC's hosting arrangements.

Governing body

Throughout this questionnaire, the term "governing body" refers to a group of people who ultimately formulate policy and direct your HIEC along with its management, irrespective of what this group is called in your HIEC.

Self-Assessment of Governance Arrangements

***20. What is the name of your HIEC’s governing body, e.g. board, executive group, steering committee?**

***21. How many members are on this group?**

***22. Is this number about right for the governing body to accomplish its objectives?**

far too many	too many	about right	too few	far too few
○	○	○	○	○

If other than about right, please explain

***23. Does your HIEC's governing body make a distinction between non-executive and executive members?**

yes	no
○	○

If so, how many non-executive and executive members does your HIEC’s governing body have?

24. If the membership of your HIEC’s governing body reflects different sectors, how many members represent each of these sectors?

NHS provider trusts

NHS commissioners

GP practices

higher education institutions

industry

charities

local government

other (please specify)

***25. Does your HIEC have a Chair?**

yes	no
○	○

If not, please explain

26. Has the Chair been chosen as an independent person?

yes ◯ no ◯

27. If the Chair has not been chosen as an independent person, which sector does he/she represent?

- ◯ NHS provider trusts
- ◯ NHS commissioners
- ◯ GP practices
- ◯ higher education institutions
- ◯ industry
- ◯ charities
- ◯ local government
- ◯ other (please specify)

28. What is the term of the Chair?

29. How was the Chair appointed?

- ◯ self-appointed
- ◯ nominated by key partners
- ◯ by consensus or vote among all partners
- ◯ through an open competitive process
- ◯ other (please specify)

30. Is the Chair paid?

yes ◯ no ◯

31. Does the Chair work a set number of hours per week?

yes ◯ no ◯

If so, how many days a week or wte?

*32. What is the frequency of governing body meetings?

Self-Assessment of Governance Arrangements

***33. Do all members of your HIEC's governing body have equal decision-making/voting rights?**

	yes	no
	◯	◯

If not, please explain

***34. Does the HIEC have any other body involved in the governance process, e.g. any advisory group, wider consultative committee, patient, or student representation?**

	yes	no
	◯	◯

If so, please explain

35. Please add any further comments you may have to explain your governance arrangements.

Decision-making authority and dynamics of the governing body

***36. Does your HIEC's governing body have authority to allocate resources?**

yes	no
◯	◯

***37. Does your HIEC's governing body have authority to establish partnership initiatives?**

yes	no
◯	◯

***38. Does your HIEC's governing body have authority to report partnership performance?**

	yes	no
back to the partner organisations	◯	◯
to the SHA	◯	◯
to the sector they represent	◯	◯

other (please specify)

***39. Does your HIEC's governing body have authority to navigate the future through transition?**

yes	no
◯	◯

***40. Do you feel that the decision-making process occurs in a politically charged atmosphere?**

strongly agree	agree	neither agree nor disagree	disagree	strongly disagree
◯	◯	◯	◯	◯

if agree or strongly agree, please explain

***41. Over your HIEC's lifetime, how often were there disagreements between or among governing body members?**

more than once a month	about once a month	once every several months	about once a year	not at all
◯	◯	◯	◯	◯

if once a month or more, please explain

***42. On average, how serious were disagreements between or among governing body members?**

extremely serious	very serious	serious	somewhat serious	not at all serious
◯	◯	◯	◯	◯

if extremely or very serious, please explain

43. Please add any further comments you may have to explain the decision-making authority and dynamics of your HIEC's governing body.

Progress on governance activities

***44. To what extent does your HIEC's governing body own a common vision and a common mission?**

not at all	rarely	variable	partly	fully
◯	◯	◯	◯	◯

Self-Assessment of Governance Arrangements

***45. To what extent does your HIEC's governing body have a clear view of partner organisation roles?**

not at all	rarely	variable	partly	fully
○	○	○	○	○

***46. To what extent does your HIEC's governing body direct partnership organisations to fulfil their responsibilities?**

not at all	rarely	variable	partly	fully
○	○	○	○	○

***47. To what extent does your HIEC's governing body own clear policies of accountability?**

not at all	rarely	variable	partly	fully
○	○	○	○	○

***48. To what extent does your HIEC's governing body own clear plans for the HIEC when the DH funding finishes?**

not at all	rarely	variable	partly	fully
○	○	○	○	○

49. Please add any further comments you may have to explain the progress your HIEC's governing body has made on governance activities.

Management

***50. Does your HIEC have paid staff to implement decisions of the governing body?**

yes	no
○	○

If so, how many staff (in wte)?

51. How are your HIEC staff members employed (in wte)?

by the HIEC directly, wte

by the hosting organisation, wte

seconded from a partner organisation, wte

self-employed, wte

mixture of all these methods of employment, wte

other (please specify)

***52. How has the Chief Executive/Managing Director been appointed?**

- ◯ self-appointed
- ◯ appointed by key members
- ◯ by consensus among all members
- ◯ through an open competitive process
- ◯ other (please specify)

53. Across the lifetime of your HIEC, please distribute 100 points between the functions below to estimate approximately how the overall time resource of your HIEC's staff has been split between the following functions. If impossible to estimate, please proceed to the next question.

strategic leadership	
external relations and communication	
operation and project management	
general administration	
analysis/data management	
research	
clinical leadership	
other (please explain)	

54. To help us gain a sense of the ways your HIEC operates, we are asking you to think of the relative contribution of different players to your HIEC's activities. Across the lifetime of your HIEC, please estimate the relative percentage contribution of your HIEC's staff, partners, and third parties (e.g. SHA, DH, independent evaluator) to the different stages of projects across the range of your HIEC activities. If impossible to estimate, please proceed to the next question.

	HIEC staff	HIEC partners	Third parties
developing a funding model			
project initiation & planning			
project delivery			
project governance, monitoring & reporting			
project evaluation			
project dissemination			

55. Please add any further comments you may have about your HIEC's management arrangements.

Funding

56. Across the lifetime of your HIEC, please distribute 100 points between the activities below to estimate approximately how the overall expenditure has been split between these activities. If impossible to estimate, please proceed to the next question.

commissioning activities through grants or contracts

direct delivery of activities

facilitation, networking, communication, and enabling

other (please describe)

***57. Across the lifetime of your HIEC, please state approximately how much core DH funding has your HIEC received, (£)?**

58. Across the lifetime of your HIEC, please estimate approximately how much funding has your HIEC received in addition to the core DH funding, (£)?

59. Across the lifetime of your HIEC, has your HIEC received funding from any of the following sources?

- ☐ membership fees from HIEC members
- ☐ project funding from HIEC partners
- ☐ project funding from the SHA
- ☐ other NHS sources (please specify)
- ☐ other non-NHS source (please specify)

If other, please specify. Also, if you can, please indicate which of these sources is the largest one.

***60. Does your HIEC have membership fees?**

	yes	no
	◯	◯

If so, what are the reasons for introducing membership fees?

61. If your HIEC does not have membership fees, what are the reasons for not seeking membership fees?

62. Is your HIEC currently in a position to be self-sustaining without DH funds?

	yes	no
	◯	◯

other (please specify)

63. Please add any further comments you may have about funding.

Accountability

***64. Across the lifetime of your HIEC, how often has your HIEC used the following when others are delivering on your behalf?**

	never	rarely	sometimes	often	always
contracts	◯	◯	◯	◯	◯
grant agreements	◯	◯	◯	◯	◯
other formal agreements (please explain below)	◯	◯	◯	◯	◯
documented project plans	◯	◯	◯	◯	◯
agreements based on trust	◯	◯	◯	◯	◯
other informal agreements (please explain below)	◯	◯	◯	◯	◯

If other, please explain

***65. Across the lifetime of your HIEC, by whom and how often have your HIEC's projects been performance-monitored?**

	never	rarely	sometimes	often	always
governing body	○	○	○	○	○
chief executive/managing director	○	○	○	○	○
project steering group	○	○	○	○	○
project lead	○	○	○	○	○
other (please explain below)	○	○	○	○	○

If other, please explain

***66. Across the lifetime of your HIEC, how often has your HIEC used the following means of enforcement and sanctions for non-performance?**

	never	rarely	sometimes	often	always
persuasion	○	○	○	○	○
peer pressure	○	○	○	○	○
managerial sanctions	○	○	○	○	○
financial sanctions	○	○	○	○	○
other (please explain below)	○	○	○	○	○

If other, please explain

***67. Across the lifetime of your HIEC, how often has your HIEC reported on performance to the following stakeholders?**

	never	rarely	sometimes	often	always
HIEC partners	○	○	○	○	○
DH	○	○	○	○	○
SHA	○	○	○	○	○
host organisation	○	○	○	○	○
patients	○	○	○	○	○
other (please explain below)	○	○	○	○	○

If other, please explain

68. What challenges and difficulties, if any, has your HIEC encountered in terms of accountability? Please specify.

69. Please add any further comments you may have to explain accountability arrangements.

Partnership termination, succession, and legacy

***70. Is your HIEC still in operation?**

yes ◯ no ◯

If so, please indicate an expected end date?

71. If your HIEC is no longer in operation, when did your HIEC formally cease its operations?

***72. What are the main reasons for your HIEC's ceasing of operation?**

- ◯ time-limited initiative
- ◯ run out of money
- ◯ invited to join a new body, e.g. AHSC, AHSN, or LETB, please explain in the box below
- ◯ functions are being replaced by a new body, please explain in the box below
- ◯ other, please explain in the box below

if other or invited to join/being replaced by a new body, please explain in this box

***73. Has your HIEC decided on its future? If so, what is it?**

- ◯ termination of the HIEC partnership in its present form
- ◯ continuation of the HIEC partnership in a different form
- ◯ retention of the successful elements only
- ◯ discussions are still underway
- ◯ other (please specify)

***74. What do you see as your HIEC's principal legacy?**

75. Please add any further comments you may have to explain your HIEC's termination, succession, and legacy.

Governance challenges and success factors

***76. What have been the significant governance challenges for your HIEC?**

***77. What have been the significant governance success factors for your HIEC?**

78. What recommendations would you give to other cross-sector health partnerships in terms of developing and managing successful governance arrangements?

Confidentiality & further study

***79. Please note that all information you provide will be aggregated for purposes of analysis so that individual HIECs will not be identified or identifiable, unless you grant permission to cite examples from your HIEC. If you grant permission to cite at this stage and we cite examples from your HIEC in a draft report, we will share it with you first for your approval. Please indicate your preference**

OK to cite	do not cite
○	○

***80. Are you willing to discuss the issues mentioned in this survey further through interview?**

yes, I am happy to be approached for interview	no, thank you
○	○

***81. Would you be happy for a shorter version of this questionnaire to be also sent to your Chair?**

yes	no
○	○

If so, please supply their name and email

82. If so, do you think they are likely to complete the questionnaire?

probably	probably not
○	○

83. Please add any further comments or suggestions you may have about this survey.

Thank you for participating in the survey. Your inputs are valuable and much appreciated.
