# Supplementary material for: Implementation of collaborative governance in cross-sector innovation and education networks: evidence from the National Health Service in England
Source: BMC Health Serv Res. 2014 Nov 8;14:552. doi: 10.1186/s12913-014-0552-y (PMC4263053; doi:10.1186/s12913-014-0552-y)
Supplement: Additional file 2: — Survey results. [file 12913_2014_552_MOESM2_ESM.pdf]

Additional file 2: Survey results

Partnership composition	N	%	Mean	Range
4. Does your HIEC have a fixed number of partners?	17			
yes	9	53%	27	5−60
no	8	47%		
5. How have the majority of your HIEC partners been selected?	17			
self-selected (foundational members)	8	47%		
by invitation	8	47%		
by request	1	6%		
6. Does your HIEC have different classes of partners, e.g. full and affiliates?	17			
yes	5	29%		
no	12	71%		
7. Across the lifetime of your HIEC, please estimate approximately how many different organisations from each of these sectors have participated in collaborative projects initiated and supported through your HIEC:	15			
NHS provider trusts	15	100%	10	2− 40
NHS commissioners	14	100%	4	1−20
GP practices	8	53%	30	2−200
higher education institutions	15	100%	5	2−13
industry	10	67%	5	1−38
charities	10	67%	4	1−20
local government	9	60%	2	1−11
other	6	40%		
8. Across the lifetime of your HIEC, please indicate whether you feel that participation from each of these sectors has been sufficient for your HIEC to accomplish its objectives (far too little=1, too little=2, about right=3, too much=4, far too much=5):	17			
NHS provider trusts			2.94	
NHS commissioners			2.24	
GP practices			2.00	
higher education institutions			2.88	
industry			2.41	
charities			2.53	
local government			1.94	

Legal form	N	%	Mean	Range
10. Does your HIEC have a signed membership agreement or memorandum of understanding?	17			
yes	7	41%		
no	10	59%		
11. Is your HIEC registered as a charity?	17			
yes	0	0%		
no	17	100%		
12. What is the incorporation status of your HIEC?	17			
incorporated	0	0%		
unincorporated	17	100%		
17. Which sector does your HIEC's host organisation represent?	17			
NHS provider trust	5	29%		
NHS commissioners	1	6%		
higher education institutions	8	47%		
industry	1	6%		
other	2	12%		

Governing body	N	%	Mean	Range
21. How many members are on this group?	17		14	5−40
22. Is this number about right for the governing body to accomplish its objectives?	17			
far too many	0	0%		
too many	1	6%		
about right	16	94%		
too few	0	0%		
far too few	0	0%		
23. Does your HIEC's governing body make a distinction between non-executive and executive members?	17			
yes	4	24%		
no	13	76%		
24. If the membership of your HIEC's governing body reflects different sectors, how many members represent each of these sectors?	16			
NHS provider trusts	14	88%	4	1−13
NHS commissioners	9	56%	1	1−6
GP practices	3	19%	0	1−2
higher education institutions	15	94%	3	2−6
industry	6	38%	1	1−7
charities	6	38%	1	1−3
local government	5	31%	1	1−3
other	8	50%	1	
25. Does your HIEC have a Chair?	17			
yes	17	100%		
no	0	0%		
26. Has the Chair been chosen as an independent person?	17			
yes	9	53%		
no	8	47%		
27. If the Chair has not been chosen as an independent person, which sector does he/she represent?	8			
NHS provider trusts	4	50%		
higher education institutions	4	50%		
29. How was the Chair appointed?	17			
nominated by key partners	11	65%		
by consensus or vote among all partners	5	29%		
through an open competitive process	1	6%		
30. Is the Chair paid?	17			
yes	4	24%		
no	13	76%		
31. Does the Chair work a set number of hours per week?	17			
yes	0	0%		
no	17	100%		
33. Do all members of your HIEC's governing body have equal decision-making/voting rights?	17			
yes	13	76%		
no	4	24%		
34. Does the HIEC have any other body involved in the governance process, e.g. any advisory group, wider consultative committee, patient, or student representation?	17			
yes	7	41%		
no	10	59%		

Decision-making authority and dynamics of the governing body	N	%	Mean	Range
36. Does your HIEC's governing body have authority to allocate resources?	17			
yes	17	100%		
no	0	0%		
37. Does your HIEC's governing body have authority to establish partnership initiatives?	17			
yes	16	94%		
no	1	6%		
38. Does your HIEC's governing body have authority to report partnership performance?	17			
back to the partner organisations				
yes	15	88%		
no	2	12%		
to the SHA				
yes	17	100%		
no	0			
to the sector they represent				
yes	14	82%		
no	3	21%		
39. Does your HIEC's governing body have authority to navigate the future through transition?	17			
yes	12	71%		
no	5	29%		
40. Do you feel that the decision-making process occurs in a politically charged atmosphere?	17			
strongly disagree	1	6%		
disagree	7	41%		
neither agree nor disagree	7	41%		
agree	2	12%		
strongly agree	0	0%		
41. Over your HIEC's lifetime, how often were there disagreements between or among governing body members?	17			
more than once a month	0	0%		
about once a month	0	0%		
once every several months	3	18%		
about once a year	4	24%		
not at all	10	59%		
42. On average, how serious were disagreements between or among governing body members?	17			
extremely serious	0	0%		
very serious	1	6%		
serious	0	0%		
somewhat serious	1	6%		
not at all serious	15	88%		

Progress on governance activities	N	%	Mean	Range
44. To what extent does your HIEC's governing body own a common vision and a common mission?	17			
fully	10	59%		
partly	4	24%		
variable	3	18%		
rarely	0	0%		

not at all	0	0%		
45. To what extent does your HIEC's governing body have a clear view of partner organisation roles?	17			
fully	7	41%		
partly	7	41%		
variable	3	18%		
rarely	0	0%		
not at all	0	0%		
46. To what extent does your HIEC's governing body direct partnership organisations to fulfil their responsibilities?	17			
fully	8	47%		
partly	4	24%		
variable	2	12%		
rarely	2	12%		
not at all	1	6%		
47. To what extent does your HIEC's governing body own clear policies of accountability?	17			
fully	6	35%		
partly	4	24%		
variable	6	35%		
rarely	0	0%		
not at all	1	6%		
48. To what extent does your HIEC's governing body own clear plans for the HIEC when the DH funding finishes?	17			
fully	9	53%		
partly	4	24%		
variable	2	12%		
rarely	0	0%		
not at all	2	12%		

Management	N	%	Mean	Range
50. Does your HIEC have paid staff to implement decisions of the governing body? If so, how many staff (whole time equivalent)?	17			
yes	17	100%	3.7	0.2−9.9
no	0	0%		
51. How are your HIEC staff members employed (in wte)?	16			
by the HIEC directly	5	31%	0.8	1.0−3.0
by the hosting organisation	14	88%	2.5	0.1−8.0
seconded from a partner organisation	8	50%	0.5	0.5−2.0
self-employed	2	13%	0.1	0.1−1.1
mixture of all these methods of employment	1	6%	0.2	
other	1	6%	0.0	
52. How has the Chief Executive/Managing Director been appointed?	17			
appointed by key members	4	24%		
by consensus among all members	3	18%		
through an open competitive process	9	53%		
other	1	6%		
53. Across the lifetime of your HIEC, please distribute 100 points between the functions below to estimate approximately how the overall time resource of your HIEC's staff has been split between the following functions:	14			
strategic leadership	14	100%	18.0	
external relations and communication	13	93%	18.9	

operation and project management	14	100%	36.6	
general administration	11	79%	9.6	
analysis/data management	8	57%	4.5	
research	4	29%	8.1	
clinical leadership	5	36%	2.9	
other	3	21%	1.4	

**Funding**	**N**	**%**	**Mean**	**Range**
56. Across the lifetime of your HIEC, please distribute 100 points between the activities below to estimate approximately how the overall expenditure has been split between these activities:	16			
commissioning activities through grants or contracts			39.1	0−80
direct delivery of activities			34.6	0−85
facilitation, networking, communication, and enabling			25.1	1−60
other			1.3	0−10
57. Across the lifetime of your HIEC, please state approximately how much core DH funding has your HIEC received, (£ million)?	17		1.2	0.3−2.0
58. Across the lifetime of your HIEC, please estimate approximately how much funding has your HIEC received in addition to the core DH funding, (£ million)?	17		0.5	0−2.0
59. Across the lifetime of your HIEC, has your HIEC received funding from any of the following sources?	14			
membership fees from HIEC members	3	21%		
project funding from HIEC partners	5	36%		
project funding from the SHA	9	64%		
other NHS sources	6	43%		
other non-NHS source	7	50%		
60. Does your HIEC have membership fees?	17			
yes	3	18%		
no	14	82%		
62. Is your HIEC currently in a position to be self-sustaining without DH funds?	17			
yes	2	12%		
no	15	88%		

**Accountability**	**N**	**%**	**Mean**	**Range**
64. Across the lifetime of your HIEC, how often has your HIEC used the following when others are delivering on your behalf (never=1, rarely=2, sometimes=3, often=4, always=5)?	17			
contracts			2.5	
grant agreements			3.0	
other formal agreements			1.8	
documented project plans			4.1	
agreements based on trust			2.2	
other informal agreements			1.7	
65. Across the lifetime of your HIEC, by whom and how often have your HIEC's projects been performance-monitored (never=1, rarely=2, sometimes=3, often=4, always=5)?	17			
governing body			4.7	
chief executive/managing director			4.2	
project steering group			3.9	
project lead			4.8	

	N	%	Mean	Range
other			3.2	
66. Across the lifetime of your HIEC, how often has your HIEC used the following means of enforcement and sanctions for non-performance?	17			
persuasion			3.7	
peer pressure			2.7	
managerial sanctions			2.1	
financial sanctions			2.2	
67. Across the lifetime of your HIEC, how often has your HIEC reported on performance to the following stakeholders (never=1, rarely=2, sometimes=3, often=4, always=5)?	17			
HIEC partners			4.2	
Department of Health			2.8	
Strategic Health Authority			3.9	
host organisation			3.7	
patients			2.0	

Partnership termination, succession, and legacy	N	%	Mean	Range
70. Is your HIEC still in operation?	17			
yes	15	88%		
no	2	12%		
72. What are the main reasons for your HIEC's ceasing of operation?	17			
time-limited initiative	6	35%		
run out of money	1	6%		
invited to join a new body	5	29%		
functions are being replaced by a new body	4	24%		
other	1	6%		
73. Has your HIEC decided on its future? If so, what is it?	17			
termination of the HIEC partnership in its present form	5	29%		
continuation of the HIEC partnership in a different form	3	18%		
retention of the successful elements only	3	18%		
discussions are still underway	6	35%		
